# Histopathological and Reproductive Evaluation in Male Rats Fed* Jatropha curcas* Seed Cake with or without Alkaline Hydrolysis and Subjected to Heat Treatment

**DOI:** 10.1155/2017/6123408

**Published:** 2017-05-23

**Authors:** Laiane Teixeira Sousa Moura, Domenica Palomaris Mariano Souza, Simone Mendonça, José Antônio de Aquino Ribeiro, Luciano Fernandes Sousa, Adriano Tony Ramos, Paulo César Maiorka, Vera Lúcia de Araújo, Viviane Mayumi Maruo

**Affiliations:** ^1^School of Veterinary Medicine and Animal Sciences, Federal University of Tocantins, 77804-970 Araguaína, TO, Brazil; ^2^Biological Sciences, Federal University of Tocantins, 77824-838 Araguaína, TO, Brazil; ^3^Embrapa Agroenergia, 70770-901 Brasília, DF, Brazil; ^4^Department of Biological and Veterinary Sciences, Federal University of Santa Catarina, 895520-000 Curitibanos, SC, Brazil; ^5^School of Veterinary Medicine and Animal Sciences, Federal University of São Paulo, 05508-270 São Paulo, SP, Brazil

## Abstract

*Jatropha curcas* cake, a by-product of biodiesel production, is rich in protein and has potential to be used in livestock feed; however, the presence of antinutritional factors and phorbol esters limits its use. Thus, this study investigated toxicological and reproductive effects in male Wistar rats after subchronic exposure to* J. curcas* cake subjected to detoxification procedures. Rats were divided into seven groups (*n* = 10) and treated for 60 days. The control group received commercial feed, while experimental groups received a diet containing 5%* J*.* curcas* cake nonhydrolyzed or hydrolyzed with 5 M NaOH. The cakes were unwashed or washed with ethanol or water and were autoclaved at 121°C for 30 minutes. Alkaline hydrolysis combined with ethanol washing decreased the phorbol ester concentration in the cake by 98%. Histopathological findings included diffuse degeneration of the liver and edema around the pulmonary vessels in the nonhydrolyzed groups. In addition, nontreated females mated with males of nonhydrolyzed unwashed group showed a decreased number of live fetuses and an increased placental weight. There were no signs of toxicity in rats given hydrolyzed cakes washed and unwashed, indicating that alkaline hydrolysis associated with heat treatment is an efficient method for detoxification of the* J. curcas* cake.

## 1. Introduction

*Jatropha curcas* is a plant from the Euphorbiaceae family, known commonly as physic nut, that has been extensively considered for biofuel production in Brazil [1–3]. India, China, and some countries in Africa have encouraged its cultivation for biodiesel production [4–6]. Seed cake, a by-product obtained after oil extraction, possesses crude protein values ranging from 24.6% to 89% in the protein isolate [7, 8].

Despite having high nutritional value,* J. curcas* cake possesses antinutritional and toxic factors, such as curcin and phorbol esters, that limit the use of the cake in food [9]. Phorbol esters are molecules derived from tetracyclic diterpenes that are produced by plants of the Euphorbiaceae and Thymelaeaceae families and have been identified as the major toxic agent of* J. curcas* [9, 10]. Phorbol esters are cocarcinogens and induce a wide range of biological effects at low concentrations; the toxic mechanism is complex and has primarily been studied with regard to the activation of protein kinase C (PKC). PKC is involved in numerous physiological processes and plays a critical role in the signal transduction pathways that regulate cell growth and differentiation. Moreover, phorbol esters demonstrate inflammatory activity via phospholipid mobilization and the release of arachidonic acid, causing the secretion of prostaglandins and leukotrienes that trigger inflammatory responses in tissues [10].

The toxicity of* J. curcas* has been studied in different animal models, including goats, sheep, mice, rats, and carp fed with the plant [11–15]. The seeds of the plant are also toxic to humans; they have been shown to induce vomiting and diarrhoea [16].

In spite of extensive toxicological studies, little is known about the reproductive toxicity of* J. curcas* by-products. Previous studies have shown that the methanol, petroleum ether, and dichloromethane extracts of the plant fruit may cause fetal resorption when administered to females [17, 18]. In male rats, treatment with seed meal showed a decreased number of spermatogenic cells in their seminiferous tubules, congestion in the testicular blood vessels, and intertubular edema [19]. However, the reproductive effects in male rats treated with the plant and mated with untreated female rats are not established.

Several* J. curcas* seed cake detoxification procedures have been studied for inactivation of its antinutritional and toxic factors [14, 20–26]. Nevertheless, toxicity of chemical residues treatment and phorbol esters is usually not tested in the animal.

With the increasing exploitation of* J*.* curcas* as a source for biodiesel production, much residue will be generated, and its toxicological aspects must be evaluated. Therefore, the aim of this study was to investigate toxicological and reproductive effects in male rats after subchronic exposure to a diet containing* J. curcas* cake subjected to different detoxification methods.

## 2. Materials and Methods

### 2.1. Animals

For this study, 70 male and 70 female adult Wistar rats with an average weight of 270 to 350 g were obtained from the vivarium of the School of Veterinary Medicine and Animal Science of the Federal University of Tocantins. The animals were housed individually in a polyethylene cage with a metal lid (40 × 50 × 20 cm^3^), under conditions of controlled temperature (22°C to 24°C), and under a 12 : 12 light/dark hour cycle, with lights on at 6:00 am. Water and feed were supplied ad libitum throughout the entire period. All experimental procedures used in this study were maintained in accordance with the Ethics Committee on Animal Use (CEUA) of Federal University of Tocantins (UFT), Tocantins, Brazil.

### 2.2. Detoxification Procedures

*Jatropha curcas* seed cake was obtained from a biofuel production plant located in Paraíso city in the State of Tocantins, Brazil.

A method similar to that of Usman et al. [27] was adopted for alkaline hydrolysis of the cake. A total of 10 kg* Jatropha curcas* cake was divided into two portions of 5 kg each. One portion was subjected to alkaline hydrolysis and the other portion was nonhydrolyzed. The hydrolysis was carried out using NaOH 5 M (CINÉTICA®), diluted in 8 L of distilled water, and immediately homogenized with 5 kg of the ground cake. The hydrolyzed material was homogenized in a polyethylene container and then spread on aluminum trays to complete hydrolysis before being divided into three portions. One portion was washed with 92% ethanol (II), washed with distilled water (III), or unwashed (IV). The other portion of nonhydrolyzed cake also divided into three portions. One portion was washed with 92% ethanol (V), washed with distilled water (VI), or unwashed (IV). The washing procedure consisted of immersing 300 g of the hydrolyzed cake in distilled water or ethanol (300 g/L) for 30 minutes to remove excess soluble alkalis and tannins, filtering through a cotton cloth. Then, all cakes were autoclaved at 121°C for 30 minutes. The hydrolyzed and nonhydrolyzed samples were placed in an oven at 55°C for 72 hours. After cooling at room temperature, the samples were ground in a stationary mill (Willye-type TE-650®) with a 1 mm sieve and subsequently stored in a freezer until use.

### 2.3. Isolation and Quantification of Phorbol Esters

Phorbol esters were isolated according to the method described by Makkar et al. [21]. Briefly, 4 g of* J. curcas* cake was transferred to a cell in an ASE 350 accelerated solvent extractor (Dionex Corporation®) and extracted at 60°C using methanol. The extract was concentrated under nitrogen flow in a 60°C heat block. The oil residue was suspended in methanol and centrifuged, and the clear supernatant was quantitatively diluted to 5 mL with the same solvent.

The methanol extract was filtered and injected into a high performance liquid chromatography system (ProStar, Varian Inc.®) using a Zorbax SB-C18 (250 × 4.6 mm, 5 *µ*m, Agilent Technologies®) column maintained at 40°C, 0.1% phosphoric acid, an acetonitrile elution gradient at 1.1 mL/min, and ultraviolet detection at 280 nm. Phorbol 12-myristate 13-acetate (Sigma-Aldrich®) was used as an external quantification standard.

### 2.4. Administration of* Jatropha curcas* Seed Cake

Every week regular powdered chow was milled and mixed with 5% of* J. curcas* cake hydrolyzed or nonhydrolyzed, pelletized, and stored in paper bags in a temperature-controlled room (22–25°C).

Male rats were randomly distributed into 7 groups of 10 animals each. The control group (I) received commercial feed. The experimental groups received commercial feed containing 5%* J. curcas *cake nonhydrolyzed washed with ethanol (II), nonhydrolyzed washed with water (III), or nonhydrolyzed left unwashed (IV) or* J. curcas* cake hydrolyzed washed with ethanol (V), hydrolyzed washed with water (VI), or hydrolyzed left unwashed (VII), during the 60-day experimental period. The concentration of 5%* J. curcas *cake was selected to avoid nutritional disturbances. Food and water intake as well as animal weight were measured throughout the experiment.

### 2.5. Reproductive Evaluation

Untreated female rats in estrus were mated overnight with treated male rats. Mating was confirmed by the presence of spermatozoa in vaginal swabs the following morning and considered gestation day 0 (GD0). On GD20, the female rats were euthanized with intraperitoneal injections of ketamine (90 mg/kg) and xylazine (10 mg/kg). Ovaries were removed, and the number of corpora lutea was determined. Uterine horns were weighed, and the number of dead and live fetuses, the number of implantations, and the incidence of early and late resorptions in the uterine horns were recorded. A live fetus was defined as one that responds to stimuli; a dead fetus was defined as a term fetus not demonstrating marked to extreme autolysis [28]. The number of male and female fetuses was counted; for each fetus, the placenta and body weight were recorded, and fetal crown-rump length was measured. In addition, preimplantation losses were calculated using the following formula: [(number of corpora lutea − number of implants)/number of corpora lutea] × 100; postimplantation losses were calculated as follows: [(number of implants − number of viable fetuses)/number of implants] × 100 [29].

### 2.6. Organ Weight, Gross, and Histopathological Analysis

At the end of the experiment, the animals were weighed and euthanized by an intraperitoneal injection of ketamine (90 mg/kg) and xylazine (10 mg/kg) and subjected to complete necropsy. At this time, the weights of the following organs were measured: brain, heart, lung, spleen, liver, kidney, and testes. Based on the above wet weight (absolute weight) and body weight at necropsy, the relative weight was calculated. The weight of each organ was divided by the body weight and multiplied by 100 to obtain the relative organ weight (%). In addition to the above organs, samples of the thymus, adrenals, stomach, pancreas, mesenteric lymph nodes, small intestine, and large intestine were collected from each animal for histopathological examination. Samples were fixed in 10% buffered formalin, routinely embedded in paraffin, cut into 3-*µ*m-thick sections, and stained with hematoxylin and eosin (HE) for examination by light microscopy.

### 2.7. Statistical Analysis

The data were analyzed by analyses of variance (ANOVA) using the Statistical Analysis Software Sisvar® and differences between the experimental groups and the control were assessed by the Dunnett test. Fisher's test was used to compare the hydrolyzed and nonhydrolyzed groups.

Reproductive parameters were examined for normal distributions and homogeneity of variance; where relevant, the data were transformed by logarithmic function. In cases where a normal distribution and homogeneity of variance could not be obtained by data transformation, a nonparametric Kruskall–Wallis test was used, followed by the Conover test [30] for pair-wise comparisons. The level of significance for all tests was set at *P* < 0.05.

## 3. Results

### 3.1. Phorbol Ester Concentrations

**Figure 1:**
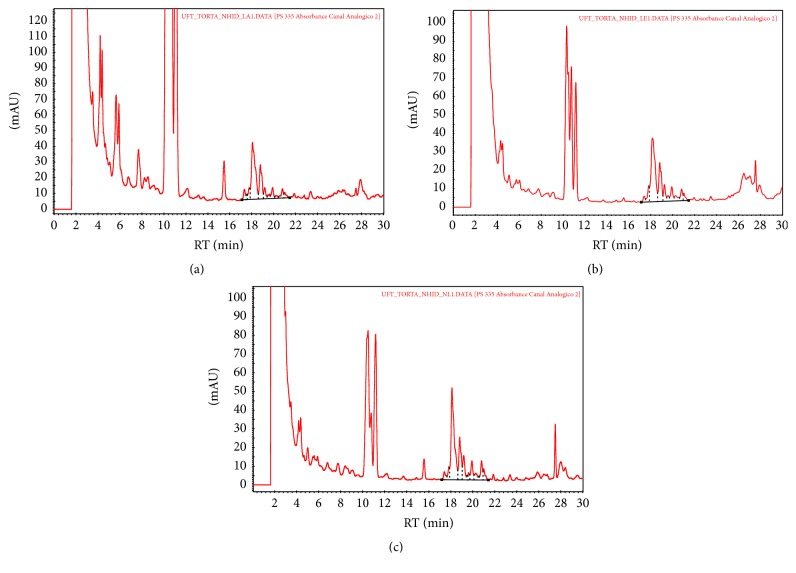
HPLC chromatograph of phorbol esters of nonhydrolyzed* J. curcas* cake washed with water (a), nonhydrolyzed cake washed with ethanol (b), and unwashed nonhydrolyzed cake (c).

**Figure 2:**
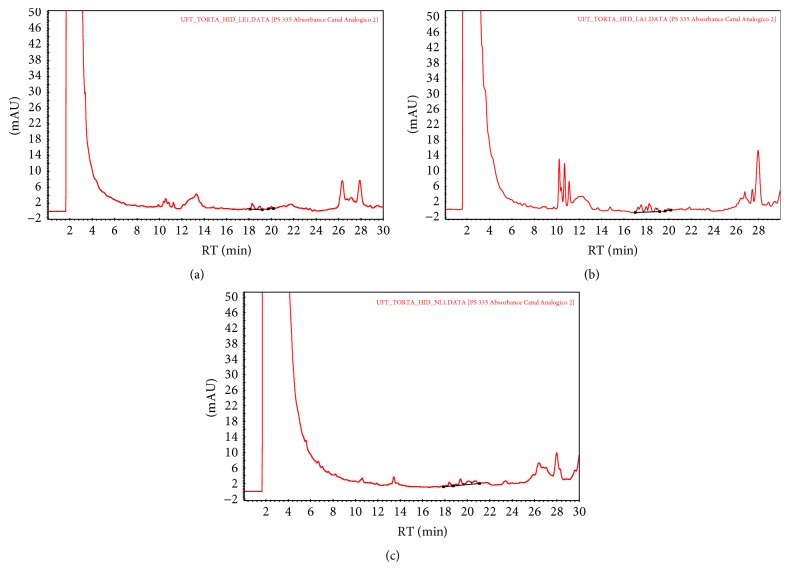
HPLC chromatograph of phorbol esters of hydrolyzed* J. curcas* cake washed with ethanol (a), hydrolyzed cake washed with water (b), and unwashed hydrolyzed cake (c).

The phorbol ester peaks showed a retention time of 17 to 21.5 min (Figures 1 and 2) and were compared to a PMA (phorbol 12-myristate 13-acetate) standard. The samples subjected to alkaline hydrolysis demonstrated a significant decrease in phorbol ester concentration. The decrease was more pronounced for the hydrolyzed* J. curcas* cake was washed with ethanol (V), which resulted in a concentration of 0.02 mg phorbol ester/g of* J. curcas* cake (Table 1).

### 3.2. Subchronic Effects in Male Wistar Rats Fed* Jatropha curcas* Seed Cake Subjected to Heat Treatment and Different Methods of Detoxification

**Figure 3:**
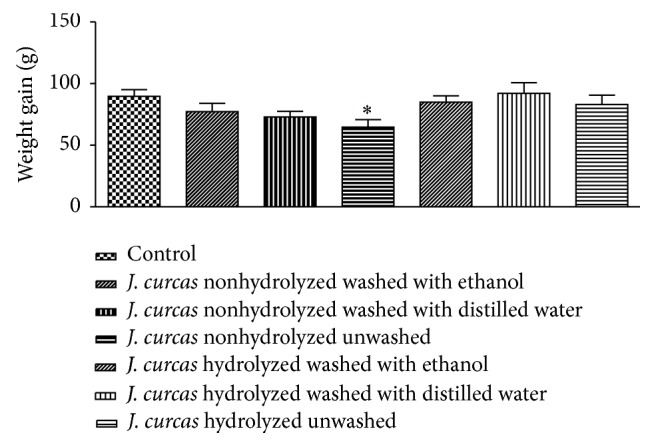
Weight gain of male Wistar rats that received a diet of 5%* J. curcas* cake subjected to heat treatment and different methods of detoxification or standard feed (control) during 60 days of the experiment. The data are expressed as the mean ± SEM. ^*∗*^*P* < 0.05, ANOVA followed by Dunnett.

In the present study, there was no mortality recorded within the hydrolyzed and nonhydrolyzed experimental groups during the experimental period. The daily phorbol ester dose ingested by the animals was as follows: 2.21 (II); 2.34 (III); 2.75 (IV); 0.05 (V); 0.13 (VI); and 0.15 (VII) mg/kg. No significant differences were detected in the food and water consumption of male rats treated with the* J. curcas* cake for 60 days compared to the control group; however, rats from group IV demonstrated a reduction in weight gain compared to the control group (Figure 3).

There were significant increases in the relative weights of the liver, kidneys, and brain in animals belonging to group IV (Table 2) compared to the control group. Additionally, Fisher's test was used to compare the hydrolyzed and nonhydrolyzed groups, revealing an increase in relative weight of the liver in the nonhydrolyzed groups (II, III, and IV) compared to the hydrolyzed groups.

**Figure 4:**
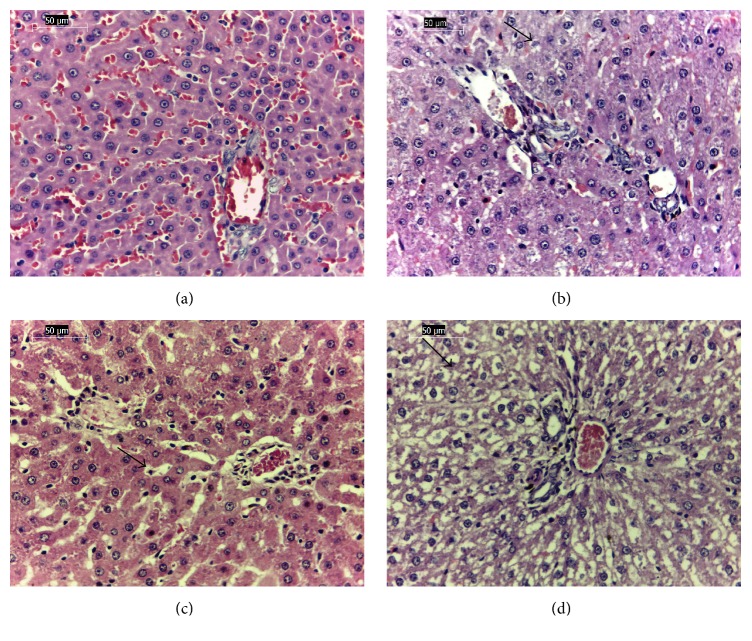
Rat liver. (a) Control; (b) liver vacuolization (arrow) in cohort fed nonhydrolyzed, ethanol-washed cake; (c) liver vacuolization (arrow) in cohort fed nonhydrolyzed, water-washed cake; and (d) liver vacuolization (arrow) in cohort fed nonhydrolyzed, unwashed cake. HE 40x.

**Figure 5:**
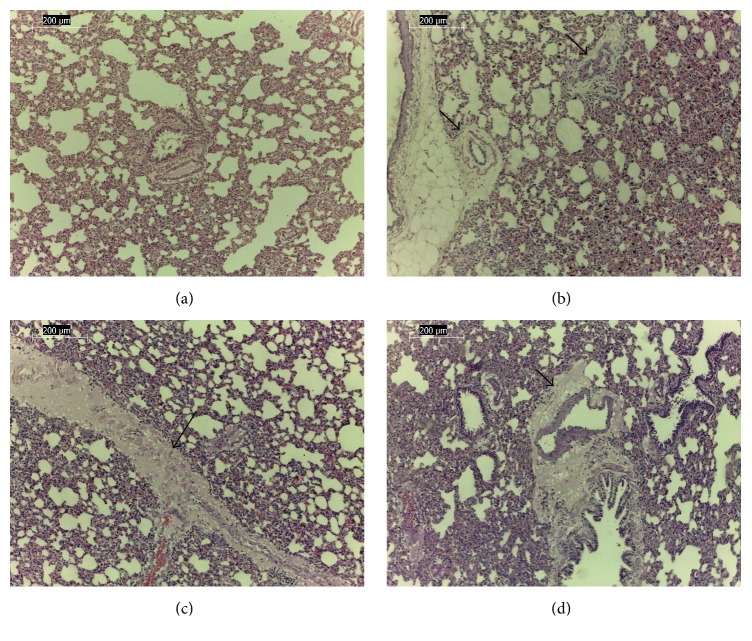
Rat lung. (a) Control; edema around the pulmonary vessels (arrow) in cohort fed nonhydrolyzed ethanol-washed cake (b); edema around the pulmonary vessels (arrow) in cohort fed nonhydrolyzed water-washed cake (c); and edema around the pulmonary vessels (arrow) in cohort fed nonhydrolyzed unwashed cake (d). HE 10x.

Histopathological findings were characterized by hepatic vacuolization in the perilobular and centrilobular areas in the animals from groups II (Figure 4(b)), III (Figure 4(c)), and IV (Figure 4(d)); other groups were not affected by treatments. The lungs of the animals from the nonhydrolyzed groups (II, III, and IV Figure 5) showed mild edema around the pulmonary vessels relative to the control group; this finding was most evident in group IV (Figure 5(d)). Other organs, such as the brain, heart, kidney, thymus, adrenals, stomach, mesenteric lymph nodes, and portions of the small and large intestines, showed normal cellular architecture.

### 3.3. Effects of* Jatropha curcas* Seed Cake Subjected to Heat Treatment and Different Methods of Detoxification on Male Reproductive Performance

**Figure 6:**
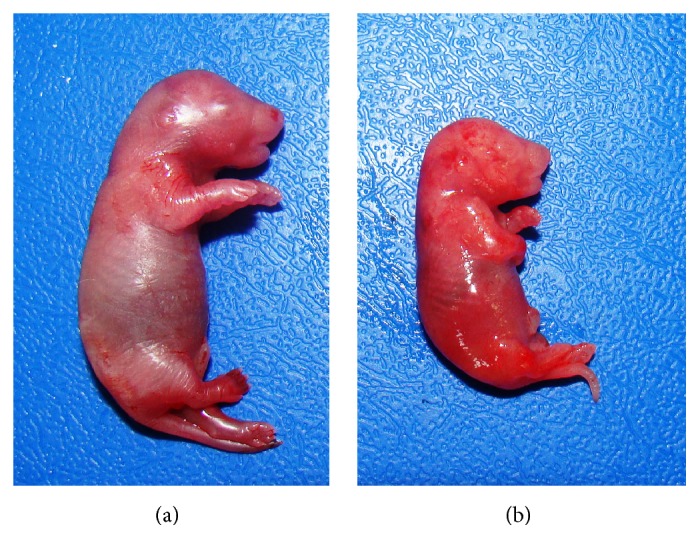
(a) Control, live fetus and (b) dead fetus in untreated female rat mated with male rat from cohort fed nonhydrolyzed unwashed cake.

No significant differences were detected in the relative weight and histological analysis of testes from rats treated with nonhydrolyzed and hydrolyzed* Jatropha curcas* cake when compared to the control group. The animals showed normal testicular architecture. By contrast, untreated female rats that were mated with male rats from group IV showed a decrease in the number of live fetuses and an increase in placental weight relative to the control group. Additionally, two stillbirths were observed in one experimental litter in this group (Table 3, Figure 6).

## 4. Discussion

The results clearly demonstrated that alkaline hydrolysis, combined with heat treatment and ethanol washing, was the most effective method in reducing the concentration of phorbol esters in* Jatropha curcas* seed cake from 1.01 to 0.02 mg/g (98%). This finding corroborates the previous reports that phorbol esters are unstable and susceptible to hydrolysis and exhibit moderate polarity, thereby allowing a greater affinity for solvents such as ethanol and methanol [10, 22, 23, 31]. No signs of toxicity were detected in the experimental groups fed* Jatropha curcas* cake treated with 5 M NaOH, heat treated, and washed with ethanol. This result confirms the efficacy of this detoxification method in removing the deleterious effects induced by the toxins present in hydrolyzed* Jatropha curcas* cake and the lack of toxic effects from chemical residues. The washing steps with water and ethanol reduced the concentrations of phorbol esters to levels of 0.89 and 0.86 mg/g (11.9% and 14.8%, resp.) in the nonhydrolyzed* J. curcas* cake; however, this reduction was insufficient to eliminate toxicity.

The toxicity and the symptoms caused by phorbol esters may vary depending on the given dosage [32]. In the present study, an intake of 2.21–2.75 mg/Kg of phorbol esters for 60 days in the groups fed the nonhydrolyzed* Jatropha curcas *seed cake (II, III, and IV) led to significant toxicity compared to animals that received hydrolyzed* Jatropha curcas* seed cake (groups V, VI, and VII). This difference was most likely due to the presence of phorbol esters because heat treatment cannot inactivate this compound [9, 14, 19].

In the current study, there was reduced body weight gain compared to the other groups and increased relative weight of the liver, kidneys, and brain of the animals from group IV as well as liver degeneration in animals from nonhydrolyzed groups that may have resulted from the higher intake of phorbol esters in the diet (2.21–2.75 mg/kg). These findings indicate a tissue dysfunction due to the direct effect of phorbol esters on organs, as reported in other studies with* J. curcas* [19, 32]. The mechanism of action for this finding is not clearly defined in the present study; however, it has been established that phorbol esters promote tissues damage by the release of cytokines and proteases and activation of NADPH oxidase [10, 33].

Moreover, phorbol esters disrupt the integrity of the endothelial cell monolayer and cause intercellular gap formation, promoting vascular permeability in vitro and lung edema in vivo [34, 35].

Experiments evaluating reproductive toxicity are generally performed to determine the direct effects of a chemical on mammalian reproduction [36]. Treatment of only males with a variety of agents has been shown to produce adverse effects in offspring, including pre- and postimplantation loss [37]. Historically, the average preimplantation loss in developmental toxicity studies is 7.6%, while the average postimplantation loss in developmental toxicity and natural delivery studies is 6.0 and 8.8%, respectively [29]. In fact, in the current study, the females mated with animals from group IV demonstrated a higher rate of pre- and postimplantation losses than the historic expected average.

The decrease in the number of live fetuses and increase in placental weight, observed in untreated female rats that were mated with male rats from group IV, may be related to the action of phorbol esters on paternal DNA. Phorbol ester-induced generation of reactive oxygen species (ROS) could directly damage sperm DNA, compromising the paternal genome. Additionally, spermatozoa with DNA damage could lead to defects in embryo development [38–41].

## 5. Conclusion

Our results demonstrate that the 5 M NaOH treatment, followed by heat treatment and minimal washing with ethanol for a short duration (30 min), was most effective in reducing phorbol esters in the* J. curcas* cake. Furthermore, the animals belonging to the cohorts fed hydrolyzed cake, either washed with water or unwashed, presented no signs of toxicity; these findings indicate that alkaline hydrolysis alone was sufficient to detoxify the cake. Although water washes remove alkali excess, the data demonstrate that additional washes after hydrolysis are not necessary. Alkaline hydrolysis is a low-cost methodology that could be used in regions with intense dry seasons to detoxify* J. curcas* cake, thereby allowing its use as a nutritious alternative protein source in livestock feed.

## Figures and Tables

**Table 1:** Concentration of phorbol ester from *Jatropha curcas* seed cake subjected to heat treatment and different methods of detoxification.

*J. curcas* cake	Phorbol esters (mg/g)
Nonhydrolyzed washed with ethanol (II)	0.86
Nonhydrolyzed washed with water (III)	0.89
Nonhydrolyzed unwashed (IV)	1.01
Hydrolyzed washed with ethanol (V)	0.02
Hydrolyzed washed with water (VI)	0.05
Hydrolyzed unwashed (VII)	0.06

**Table 2:** Relative organ weight of male rats that received a diet of 5% *Jatropha curcas* seed cake subjected to heat treatment and different methods of detoxification or standard feed (control) for 60 days (*n* = 10).

Organs	Control	*J. curcas *nonhydrolyzed			*J. curcas *hydrolyzed		
	(I)	Washed with ethanol (II)	Washed with water (III)	Unwashed (IV)	Washed with ethanol (V)	Washed with water (VI)	Unwashed (VII)
Heart (%)	0.26 ± 0.01	0.27 ± 0.01	0.29 ± 0.01	0.29 ± 0.01	0.26 ± 0.01	0.26 ± 0.01	0.26 ± 0.01
Lungs (%)	0.46 ± 0.03	0.46 ± 0.04	0.49 ± 0.05	0.48 ± 0.04	0.51 ± 0.03	0.50 ± 0.05	0.45 ± 0.03
Spleen (%)	0.23 ± 0.01	0.24 ± 0.01	0.25 ± 0.01	0.24 ± 0.01	0.23 ± 0.01	0.25 ± 0.01	0.23 ± 0.01
Liver (%)	3.23 ± 0.10	3.65 ± 0.14	3.42 ± 0.15	3.76 ± 0.15^**∗**^	3.35 ± 0.14	3.34 ± 0.10	3.26 ± 0.14
Kidneys (%)	0.74 ± 0.03	0.77 ± 0.02	0.77 ± 0.04	0.84 ± 0.03^**∗**^	0.77 ± 0.01	0.76 ± 0.03	0.77 ± 0.02
Testis (%)	0.76 ± 0.03	0.83 ± 0.02	0.82 ± 0.02	0.86 ± 0.05	0.83 ± 0.02	0.86 ± 0.03	0.80 ± 0.03
Brain (%)	0.45 ± 0.01	0.46 ± 0.01	0.47 ± 0.01	0.51 ± 0.02^**∗**^	0.46 ± 0.01	0.46 ± 0.01	0.46 ± 0.01

Data shown are mean ± SEM.

^*∗*^ *P* < 0.05, ANOVA followed by Dunnett.

**Table 3:** Reproductive parameters of untreated female rats mated with male rats that received a diet of 5% of *Jatropha curcas* seed cake subjected to heat treatment and different methods of detoxification or standard feed (control) during 60 days.

Parameters	Control	*J. curcas *nonhydrolyzed			*J. curcas *hydrolyzed		
	I	Washed with ethanol (II)	Washed with water (III)	Unwashed (IV)	Washed with ethanol (V)	Washed with water (VI)	Unwashed (VII)
Pregnant total	9/10	8/10	8/10	10/10	9/10	8/10	9/10
Maternal body weight (g)^a^	360 ± 14.38	371.12 ± 11.31	377 ± 7.94	363.00 ± 8.03	351.33 ± 9.44	366.75 ± 14.15	369.44 ± 8.51
Pregnant uterus weight (g)^a^	61.57 ± 5.68	56.58 ± 5.25	62.01 ± 5.73	51.6 ± 6.24	61.10 ± 5.16	58.65 ± 6.61	61.89 ± 5.77
Number of corpora lutea^a^	13.66 ± 0.50	14.00 ± 0.60	13.25 ± 0.67	13.00 ± 0.65	13.77 ± 0.61	13.62 ± 0.56	13.55 ± 0.70
Number of implantations^a^	13.33 ± 0.55	12.50 ± 0.59	12.25 ± 0.70	11.30 ± 0.65	12.44 ± 1.04	12.37 ± 0.56	12.66 ± 0.80
Number of reabsorption sites^a^	0.44 ± 0.24 (3)	0.87 ± 0.47 (4)	0.50 ± 0.18 (4)	1.90 ± 0.67 (8)	0.88 ± 0.51 (3)	0.75 ± 0.25 (5)	0.77 ± 0.43 (3)
Placental weight (g)^a^	0.40 ± 0.02	0.43 ± 0.04	0.44 ± 0.03	0.51 ± 0.03^**∗**^	0.46 ± 0.01	0.42 ± 0.02	0.45 ± 0.02
Litter weight (g)^a^	35.24 ± 4.89	34.29 ± 4.44	35.13 ± 4.56	29.03 ± 4.73	34.63 ± 4.27	35.72 ± 7.27	35.06 ± 5.45
Body weight of fetuses (g)^a^	2.70 ± 0.21	2.98 ± 0.18	2.91 ± 0.15	3.11 ± 0.25	2.94 ± 0.19	2.91 ± 0.36	2.92 ± 0.27
Crown-rump length (cm)^a^	4.90 ± 0.21	4.76 ± 0.12	4.75 ± 0.11	4.65 ± 0.16	4.88 ± 0.11	4.84 ± 0.30	4.90 ± 0.13
Number of live fetuses^a^	12.88 ± 0.73	11.62 ± 0.82	11.75 ± 0.79	9.20 ± 0.87^**∗**^	11.55 ± 0.83	11.62 ± 0.62	11.89 ± 0.69
Number of dead fetuses^a^	0.00 ± 0.00	0.00 ± 0.00	0.00 ± 0.00	0.20 ± 0.00 (1)	0.00 ± 0.00	0.00 ± 0.00	0.00 ± 0.00
Preimplantation loss (%)^b^	2.45 ± 1.77 (2)	10.30 ± 3.73 (5)	7.36 ± 2.91 (5)	11.97 ± 5.56 (4)	10.57 ± 5.25 (4)	8.85 ± 3.31 (5)	6.53 ± 3.06 (4)
Postimplantation loss (%)^b^	3.82 ± 2.14 (3)	7.29 ± 4.00 (4)	4.50 ± 1.75 (4)	18.17 ± 6.11 (8)	5.85 ± 3.30 (3)	6.17 ± 1.94 (5)	5.47 ± 2.94 (3)

Data shown are mean ± SEM. Values in parentheses indicate the number of litters affected.

Where appropriate: ^a^Dunnett test; ^b^Kruskall-Wallis test. ^*∗*^*P* < 0.05 compared to the control group.
